# Dysregulation of lipid metabolism and pathological inflammation in patients with COVID-19

**DOI:** 10.1038/s41598-021-82426-7

**Published:** 2021-02-03

**Authors:** Marianna Caterino, Monica Gelzo, Stefano Sol, Roberta Fedele, Anna Annunziata, Cecilia Calabrese, Giuseppe Fiorentino, Maurizio D’Abbraccio, Chiara Dell’Isola, Francesco Maria Fusco, Roberto Parrella, Gabriella Fabbrocini, Ivan Gentile, Immacolata Andolfo, Mario Capasso, Michele Costanzo, Aurora Daniele, Emanuela Marchese, Rita Polito, Roberta Russo, Caterina Missero, Margherita Ruoppolo, Giuseppe Castaldo

**Affiliations:** 1 CEINGE - Biotecnologie Avanzate s.c.ar.l., Via Gaetano Salvatore, 486, 80145 Naples, Italy; 2 Dipartimento di Medicina Molecolare e Biotecnologie Mediche, Università degli Studi di Napoli “Federico II”, 80131 Naples, Italy; 3 Dipartimento di Biologia, Università degli Studi di Napoli “Federico II”, 80126 Naples, Italy; 4 Fisiopatologia e Riabilitazione Respiratoria-1 utsir COVID, Azienda Ospedaliera Specialistica dei Colli, Naples, Italy; 5 Dipartimento di Scienze Mediche Traslazionali, Università degli Studi della Campania “Luigi Vanvitelli”, 81100 Naples, Italy; 6 Dipartimento di malattie infettive ed urgenze infettivologiche, COVID Unit, Azienda Ospedaliera Specialistica dei Colli, Naples, Italy; 7 Dipartimento di Medicina Clinica e Chirurgica, Università degli Studi di Napoli, “Federico II”, 80131 Naples, Italy; 8 Dipartimento di Scienze e Tecnologie Ambientali, Biologiche, Farmaceutiche, Università degli Studi della Campania “Luigi Vanvitelli”, 81100 Naples, Italy; 9 Dipartimento di Salute Mentale e Fisica e Medicina Preventiva, Università degli Studi della Campania “Luigi Vanvitelli”, 81100 Naples, Italy; 10 Dipartimento di Sanità Pubblica, Università degli Studi di Napoli, Federico II, 80131 Naples, Italy

**Keywords:** Biochemistry, Biotechnology, Diseases, Molecular medicine

## Abstract

In recent months, Coronavirus disease 2019 (COVID-19) caused by severe acute respiratory syndrome coronavirus 2 (SARS-CoV-2) has spread throughout the world. COVID-19 patients show mild, moderate or severe symptoms with the latter ones requiring access to specialized intensive care. SARS-CoV-2 infections, pathogenesis and progression have not been clearly elucidated yet, thus forcing the development of many complementary approaches to identify candidate cellular pathways involved in disease progression. Host lipids play a critical role in the virus life, being the double-membrane vesicles a key factor in coronavirus replication. Moreover, lipid biogenesis pathways affect receptor-mediated virus entry at the endosomal cell surface and modulate virus propagation. In this study, targeted lipidomic analysis coupled with proinflammatory cytokines and alarmins measurement were carried out in serum of COVID-19 patients characterized by different severity degree. Serum IL-26, a cytokine involved in IL-17 pathway, TSLP and adiponectin were measured and correlated to lipid COVID-19 patient profiles. These results could be important for the classification of the COVID-19 disease and the identification of therapeutic targets.

## Introduction

Coronavirus disease 2019 (COVID-19) caused by severe acute respiratory syndrome coronavirus 2 (SARS-CoV-2) has spread rapidly around the world in recent months and has become an overwhelming health threat globally. COVID-19 has affected more than 16 million individuals and caused nearly 650,000 deaths, resulting in an average mortality of 4% worldwide as of July 27th, 2020, compared with the influenza mortality rate of less than 1%. 80% of patients have mild or moderate symptoms and are therefore classified as mild or moderate COVID-19^1^, whereas 20% of patients develop severe symptoms in time like respiratory distress and require immediate access to specialized intensive care and are therefore classified as clinically severe^2–6^.

Molecular bases of SARS-CoV-2 infections, pathogenesis and progression have not been clearly elucidated yet, thus forcing the development of many complementary approaches to identify candidate cellular pathways involved in disease progression. An incomplete understanding of the systemic response to SARS-CoV-2 is a major obstacle to the development of new therapeutics and a vaccine^7^. Double-membrane vesicles (DMVs)^8^ have been described to be optimal for coronavirus replication^9^ due to the unique lipid micro-environment supporting the hypothesis that host lipids play a critical role in replication cycle of viruses^8^. In addition, lipid biogenesis pathways affect receptor-mediated virus entry at the endosomal cell surface^10,11^ and modulate virus propagation^12,13^. The development of acute distress respiratory syndrome may also alter lipids implicated in inflammatory process.

In this study, we have combined targeted mass spectrometry (MS)-based lipidomics with the measurement of a subset of proinflammatory cytokines in blood serum of COVID-19 hospitalized patients. Circulating cytokines are crucially important in the systemic response to viruses. However, several studies have established that in severe COVID-19 cases massive cytokine released in the bloodstream may cause a cytokine storm, similar to the one occurring in the macrophage activation syndrome^14,15^. Such an hyperinflammatory response can contribute to systemic coagulation and fatality in infected patients. In severe cases of COVID-19 infection, an induction of reported increases in inflammatory monocytes and neutrophils and a sharp decrease in lymphocytes have been reported, with the pro- inflammatory cytokines include interleukins IL-1ß, IL-6 and IL-8, and TNF-α^16–18^.

Viral infection causes release of alarmins, also called damage- associated molecular patterns (DAMPs), by dying necrotic cells. Among the alarmins, thymic stromal lymphopoietin (TSLP) is a distant paralog of IL-7 mainly released by epithelial lining of the lungs, gastrointestinal tract and skin in response of an external insults, which when produced in high amount is released in the bloodstream^19^. In addition, it also participates to an adaptive immune response against viruses that target the respiratory mucosa^20^. Finally, in inflammatory processes triggered by viral infection adiponectin, selectively produced by adipose tissue, strongly correlates to severity of inflammation^21–23^.

In this paper, targeted metabolomic analysis was carried out to define the lipidomic serum profile of a cohort of COVID-19 patients characterized by different severity degree, following the hypothesis that SARS-CoV-2 induces metabolic changes in the body fluids, as reported very recently^24,25^. In all these patients, we previously found increased levels of serum interleukin (IL)-6 together with variable levels of tumor necrosis factor (TNF)-α, IL-17A and the serum receptor of IL-17A (IL-17RA)^26^. Here, we measured serum IL-26, a cytokine involved in IL-17 pathway, TSLP and adiponectin. Targeted lipidomics was correlated to inflammatory cytokines identifying significant correlations in infected patients. These findings may have important implications in the classification of the disease and in the identification of therapeutic targets.

## Results

### Serum lipidomics in COVID-19 patients

**Figure 1:**
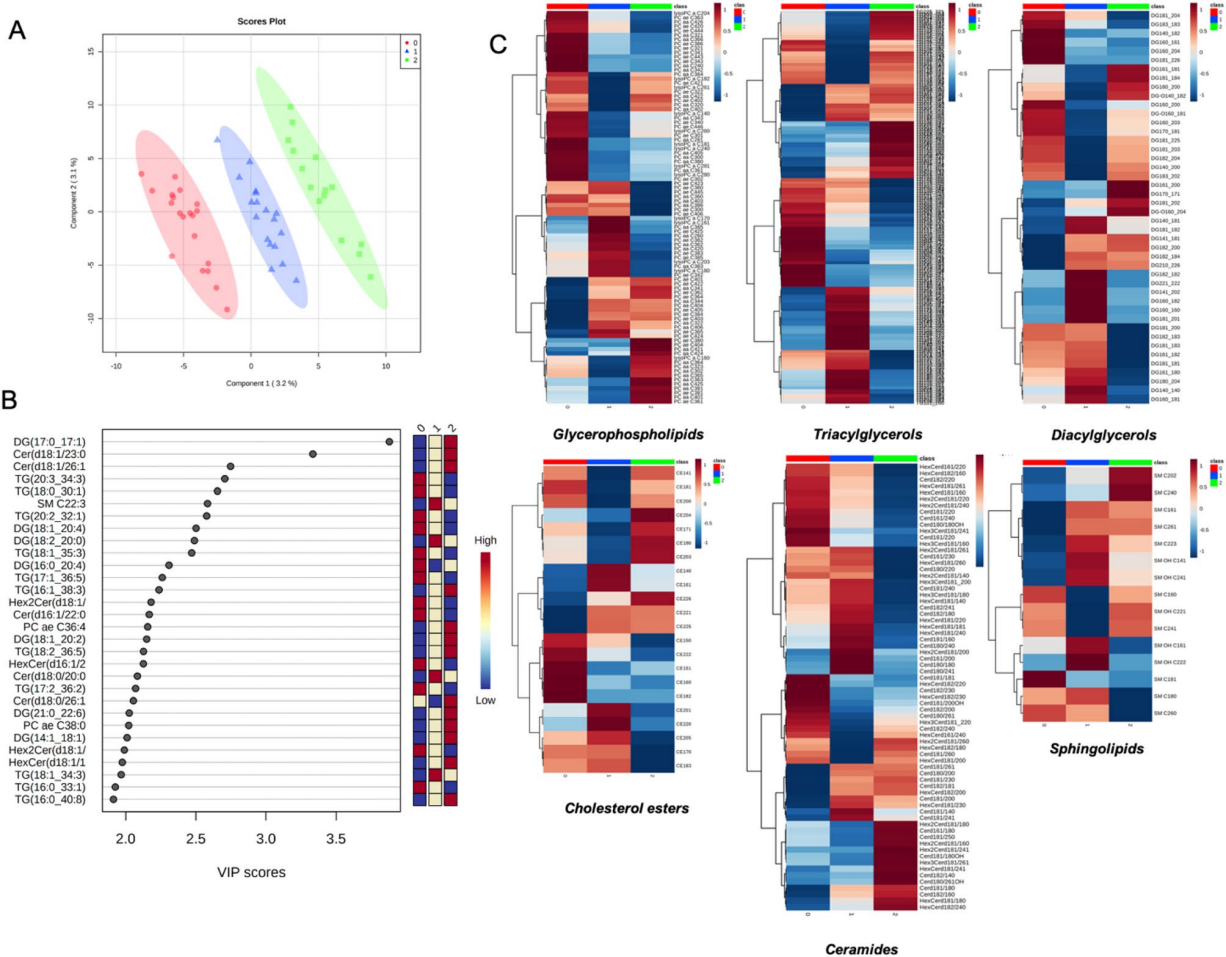
Lipid profiles in COVID-19 patient serum. (**A**) Supervised partial least squares-discriminant analysis (PLS-DA) score plot and (**B**) discriminant lipidic features according to the variable importance on projection (VIP) score of mild, moderate and severe COVID-19 patients. The top 30 important lipids (VIP score ≥ 2.0 were summarized according to their VIP score value. The intensity of the colored boxes indicates the relative lipids abundance in each group (0 mild, 1 moderate and 2 severe patients). (**C**) Heatmaps of the auto-scaled mean lipid concentrations of each patient groups (0 mild, 1 moderate and 2 severe patients) of significantly altered lipids (*p* < 0.05) in six different lipid classes. The heatmap color code represents the relative lipid abundance. The concentrations of the metabolites were imputed, log(2) transformed, and Pareto scaled.

**Figure 2:**
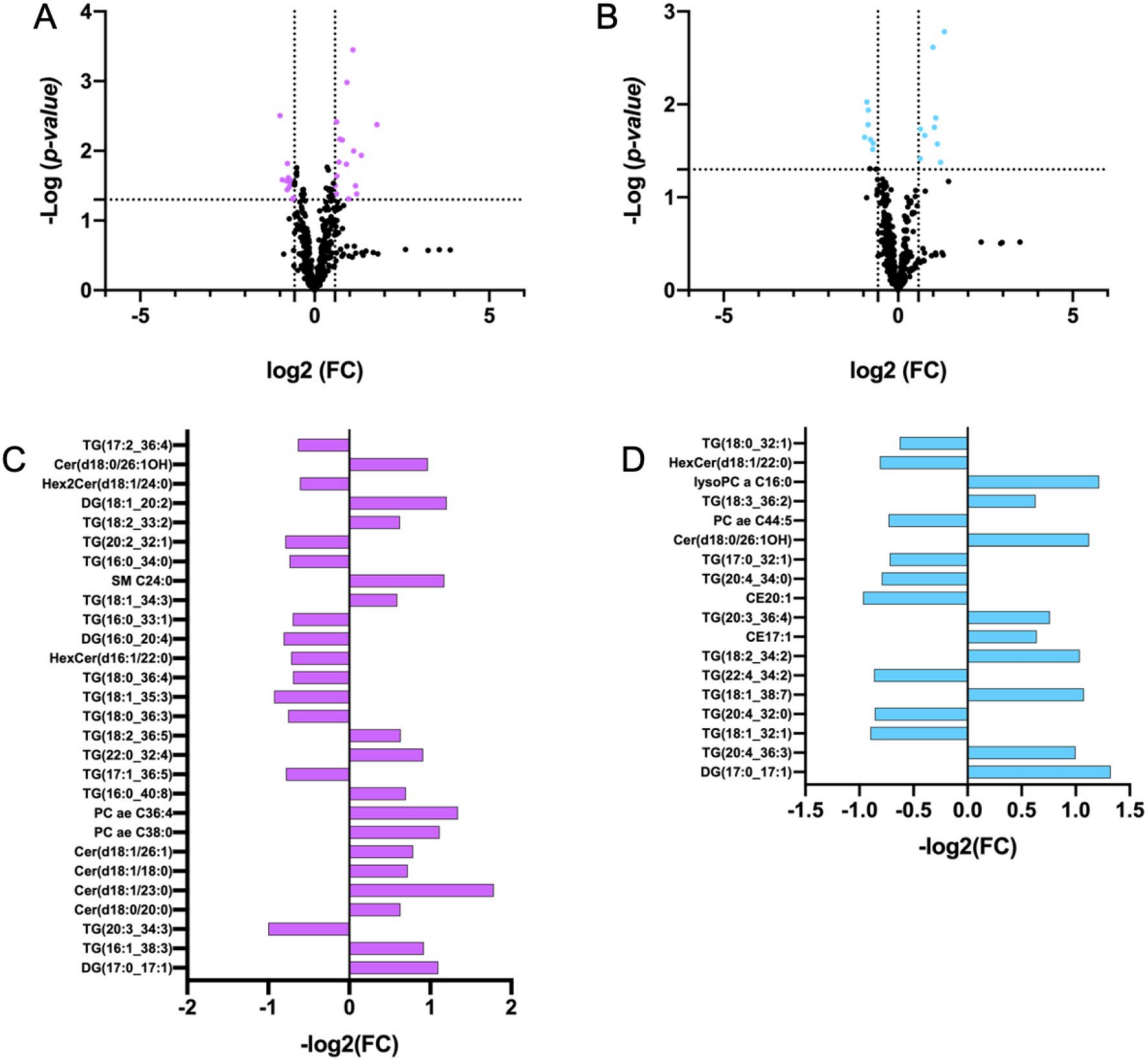
Targeted lipidomic analyses. Volcano plots of differential concentrations of 483 lipids in comparison of (**A**) severe patient serum to mild and (**B**) severe patient serum to moderate. The fold change of significant differentially abundant lipids (*p* < 0.05) in (**C**) severe condition versus mild and (**D**) severe condition versus moderate were plotted according to their significant *p-value*, reported in Tables 1 and 2, respectively.

Six classes of lipids, as diacylglycerols (DG) (n = 44 molecules), triacylglycerols (TG) (n = 242 molecules), glycerophospholipids (LPC, PC aa, PC ae) (n = 90 molecules), cholesterol esters (CE) (n = 22), ceramides (Cer) (n = 70) and sphingolipids (SM) (n = 15 molecules), were dosed. A detailed list of the measured lipids is shown in Table S1 and their concentrations in Table S2. The concentrations of the lipids from serum COVID-19 patients were imputed to remove missing value, log(2) transformed and pareto scaled; the lipid datasets, corresponding to the 483 analyzed metabolites (Table S2), were processed by partial least squares-discriminant analysis (PLS-DA) to evaluate the difference rate and variance occurring between the groups according to pathology severity. These data points were clustered into three distinct groups in the plot map (Fig. 1A), separating the COVID-19 patients according to pathology-severity, (principal component, PC1 variance 3.2%, PC2 variance 3.1%). Outliers were not found in serum lipid datasets. The most discriminant lipidic molecules between the three groups of patients, estimated according to their Variable Importance in Projection (VIP) score (threshold > 2.0) included 28 metabolites (Fig. 1B); the levels of 12 lipidic molecules, including DG(17:0_17:1), DG(18:1_20:2), DG(21:0_22:6), DG(14:1_18:1), TG(16:1_38:3), TG(18:2_36:5), TG(16:0_40:8), PC ae C36:4, PC ae C38:0, Cer(d18:1/23:0), Cer(d18:1/26:1), HexCer(18:1/18:0), increased in severe COVID19 serum in respect to moderate and mild groups and 10, including DG(18:1_20:4), TG(20:3_34:3), TG(18:0_30:1), TG(18:1_35:3), TG(17:1_36:5), TG(17:2_36:2), TG(16:0_33:1), Cer(d16:1/22:0), Hex2Cer(d18:1/24:0), Hex2Cer(d18:1/22:0), decreased in severe COVID19 serum in respect to moderate and mild groups. In order to explore the details of the observed discrimination trend, a heatmap was constructed according to the degree of biochemical features of analyzed lipids. As indicated in Fig. 1C, changed lipid features affect all the analyzed lipidic classes, indicating that virus infection strongly perturbed lipid homeostasis. Univariate analysis allows to detect significant differences in lipidic profiles when severe-, medium- and mild-patients have been compared each other (Fig. 2A,B). Severe patients displayed altered levels of DG(17:0_17:1), DG(16:0_20:4), DG(18:1_20:2), TG(16:1_38:3), TG(20:3_34:3), TG(16:0_40:8), TG(17:1_36:5), TG(22:0_32:4), TG(18:2_36:5), TG(18:0_36:3), TG(18:1_35:3), TG(18:0_36:4), TG(16:0_33:1), TG(18:1_34:3), TG(16:0_34:0), TG(20:2_32:1), TG(18:2_33:2), TG(17:2_36:4), Cer(d18:0/20:0), Cer(d18:1/23:0), Cer(d18:1/18:0), Cer(d18:1/26:1), Cer(d18:0/26:1OH), HexCer(d16:1/22:0), Hex2Cer(d18:1/24:0), PC ae C38:0, PC ae C36:4, SM C24:0, in respect to mild patients (Table 1, Fig. 2C). In particular, serum concentrations of ceramides, as Cer(d18:0/20:0), Cer(d18:1/23:0), Cer(d18:1/18:0), Cer(d18:1/26:1), Cer(d18:0/26:1OH), were significantly increased while glycosylceramides as HexCer(d16:1/22:0), Hex2Cer(d18:1/24:0), were significantly decreased in severe condition versus mild condition. Moreover, severe patient serum concentrations of PC ae C38:0, PC ae C36:4, SM C24:0, resulted increased in respect to mild patients. When the severe condition was compared to moderate condition, significant alterations in serum level of DG(17:0_17:1), TG(20:4_36:3), TG(18:1_32:1), TG(20:4_32:0), TG(18:1_38:7), TG(22:4_34:2), TG(18:2_34:2), CE17:1, TG(20:3_36:4), CE20:1, TG(20:4_34:0), TG(17:0_32:1), Cer(d18:0/26:1OH), PC ae C44:5, TG(18:3_36:2), lysoPC a C16:0, HexCer(d18:1/22:0), TG(18:0_32:1) (Table 2, Fig. 2D). The alteration in ceramide levels was conserved also when the severe condition was compared to the moderate one. Indeed, serum concentrations of ceramide Cer(d18:0/26:1OH) were significantly increased, while glycosylceramide HexCer(d18:1/22:0) decreased in severe condition versus moderate condition. Lipid metabolites such as DG(17:0_17:1), TG(20:4_36:3), TG(18:1_38:7), TG(18:2_34:2), TG(20:3_36:4), TG(18:3_36:2), CE17:1, lysoPC a C16:0, have been observed as more abundant in severe versus moderate condition, and TG(20:4_36:3), TG(18:1_32:1), TG(22:4_34:2), CE20:1, TG(20:4_34:0), TG(17:0_32:1), PC ae C44:5 and TG(18:0_32:1), resulted less abundant.

### Serum levels of cytokines and their correlation with metabolic changes

**Figure 3:**
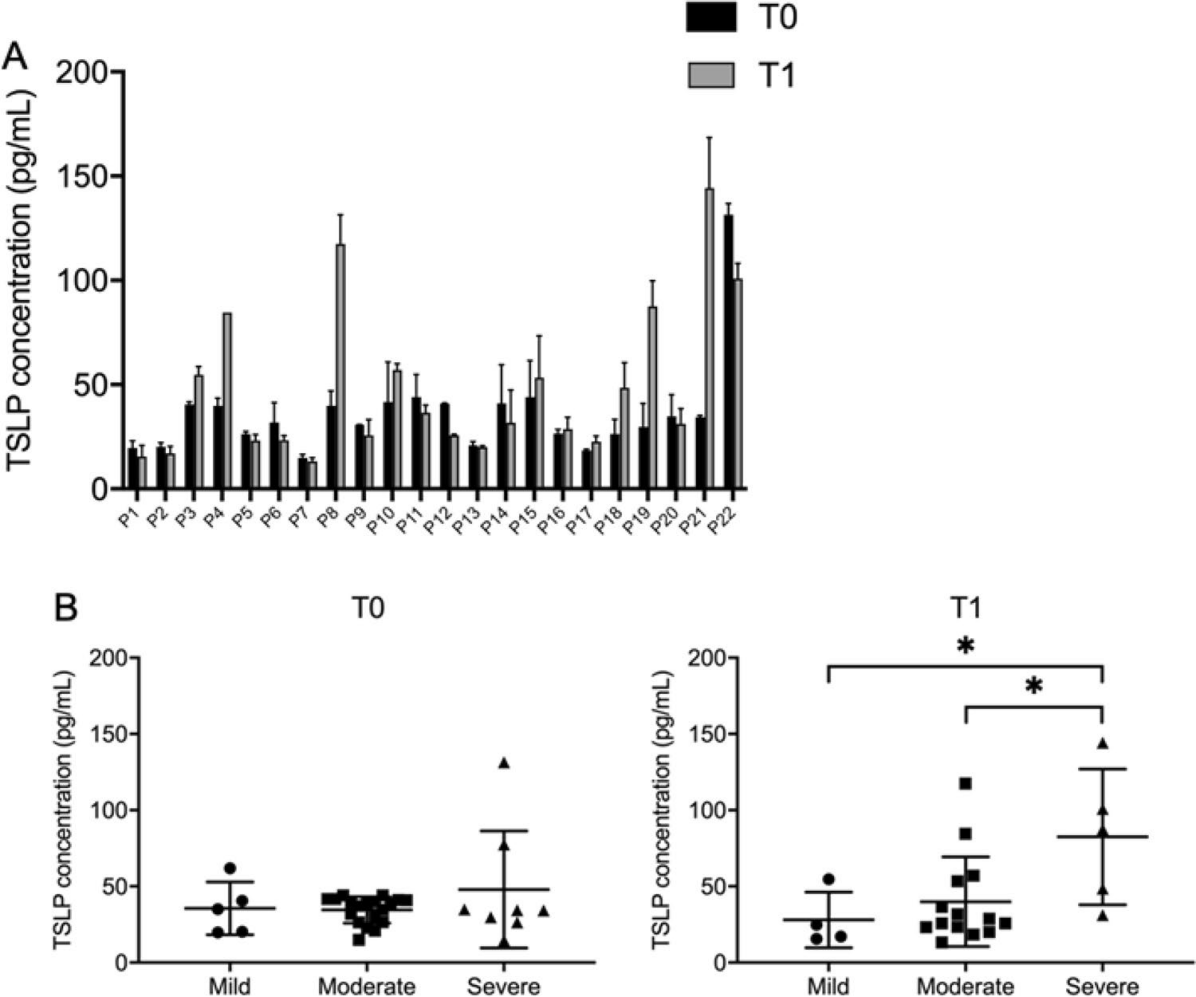
TSLP protein levels in COVID-19 patient serum. (**A**) TSLP concentration expressed in pg/mL in the serum of COVID-19 patients at time of hospital admission (T0) or 1-week after admission (T1). (**B**) TSLP levels in severely affected individuals was significantly higher than in mild or moderate cases (adjusted *p*-value = 0.048 and 0.046 respectively).

Being a lytic virus, SARS-CoV-2 causes cell death leading to local and systemic inflammation with the upregulation of several cytokines. Injured tissue releases alarmins such as TSLP that may be released in the bloodstream and have multiple effects. To test the possibility that SARS-CoV-2 infection leads to TSLP accumulation in the bloodstream, we measured the levels of serum TSLP in individuals affected by COVID-19 at two time points, namely at hospital admission and 7 days post admission. In most tested patients the TSLP serum levels remained similar or were elevated a week after admission, although this trend did not reach statistical significance (Fig. 3A). As for the other parameters, data were then clustered into three distinct groups according to pathology-severity. At admission (T0) no significant correlation in TSLP serum levels and clinical severity was observed, whereas, 7 days after hospitalization (T1) TSLP serum levels were significantly more elevated in severe versus moderate or mild cases (Fig. 3B and Table S3).

IL-26 was not correlated to the severity of the disease in this cohort (Table S3), although we found that 5 moderate patients had IL-26 concentrations from fivefold to 200-fold higher than upper reference value (10 pg/mL) and only one mild patient showed a IL-26 level higher than reference value. At admission (T0) no significant correlations were found for IL-26. 7 days after hospitalization (T1), IL-26 positively correlated with Cer(d18:0/d18:0), whereas a negative correlation was observed between IL-6 and the cholesterol ester CE(22:1) (Table S3).

### Serum level of adiponectin and its correlation with metabolic changes

Total adiponectin evaluation by ELISA-test showed that the COVID-19 patients subdivided in three sub-groups based have different adiponectin concentrations. In particular, in severe COVID-19 adiponectin levels are higher compared to moderate and mild COVID-19 patients. However, the difference between the three subgroups are not statistically significant (Table 3). Western blotting analysis showed the oligomeric profile of adiponectin in COVID-19 patients and confirmed the above described adiponectin differences (Supplementary Fig. 1). Full-length image of blot was reported in Supplementary Fig. 2. At T1 IL-6 serum levels positively correlated to adiponectin (Table S3). Furthermore, a positive correlation among adiponectin and ceramides and glycerophospholipids was found, at T0 (Table S3).

## Discussion

MS-based lipidomic platform allowed to define the lipidic profiles according to infection-pathology severity by measuring 483 lipids, included in six different lipidic classes. MS targeted lipidomic approach coupled to multivariate and univariate statistical analyses identified alterations in serum concentrations of 46 lipids when severe patients were compered to mild and moderate patients. Among them, a group TGs resulted altered in severe conditions. This data seems to indicate the involvement of autophagy mechanism as observed in other virus infection as dengue virus infection^27^. It has been shown that cellular lipid metabolism is influenced by autophagy, using lipid droplets^28^, constituted by triglycerides (TGs) and cholesterol esters^29^. The release of free fatty acids^30^, from triglycerides hydrolysis by lipases enzyme, unbalances mitochondria homeostasis and subsequent β-oxidation and energy production. In addition, eleven TGs, as TG (20:3_34:3), TG(18:0_30:1), TG(20:2_32:1), TG(18:1_35:3), TG(17:1_36:5), TG(16:1_38:3), TG(18:2_36:5), TG(17:2_36:2), TG(18:1_34:3), TG(16:0_33:1), TG(16:0_40:8), were selected in the discriminant model to accurately differentiate severe conditions. According to plasma metabolomic panel differentiating COVID-19 patients from healthy controls in a recent study^25^, we defined two lipidic panels able to discriminate into severity degree of infective pathology. In Song et al.^25^ the SM sphingolipid class was demonstrated to display progressive increase with increasing of disease severity. Specifically, we found an increased level of SM C24:0 in severe versus mild condition.The membrane composition and fluidity alteration were described as the main biological process responsible of microdomains generation, able to affect the dynamic roles of membrane, as signal transduction and immune activation processes^31^.

It is interesting to note also the significant increased level of ceramides, Cer(d18:0/20:0), Cer(d18:1/23:0), Cer(d18:1/18:0), Cer(d18:1/26:1), in serum of severe patients. Recently it was proved the ability of ceramide to promote vasoconstriction^32^. Ceramides belong to sphingolipid family, known as essential constituents of organelle and cell membranes that play an additional role in signal transductions of crucial physiological processes such as growth, differentiation, proliferation, migration, apoptosis, and cell death^33^. Production of ceramide may be implicated in pathological processes including cardiovascular and lung diseases^32^. An increasing scientific evidence revealed the correlation between circulating ceramide levels with adverse cardiovascular events such as myocardial infarction and stroke^34,35^. In addition, the accumulation of ceramides with long chains resulted to be associated with deleterious outcomes but it was not correlated to traditional plasma lipid markers of cardiovascular risk factors^36^.

In severe cases of COVID-19, a dysfunctional immune response triggers a cytokine storm that mediates widespread lung inflammation, although the players of immunological misfiring are still incompletely understood. Here, we find that the alarmin TSLP is released into the bloodstream and is significantly associated to the severity of the disease specifically in advanced stages (i.e. one week after hospital admission), in contrast to TNF alpha, IL-6, IL-17 and IL-26 for which this correlation was not observed. Our results were in line with previous finding indicating a dysregulated immune response in COVID-19 patients, especially in the most severe cases^25,26^. Indeed a negative correlation was demonstrated between PCs and IL-6^25^. In the present study a new negative correlation linking IL-6 and ceramides was observed, confirming the hypothesis of an inflammatory response as a consequence of the lipid alteration. In order to support these results, other clinical indices of systemic inflammation were investigated. Thymic stromal lymphopoietin (TSLP) is a mucosal tissue-associated cytokine that stimulates a T-helper type 2 (Th2) response in allergic diseases such as asthma and atopic dermatitis, and therefore plays a key role in the pathogenesis of inflammatory diseases^19^. In the human asthmatic lung TSLP is highly expressed and activates dendritic cells to strongly induce proallergic Th2 cell responses. Interestingly, a TSLP involvement in viral infection has been less investigated, although it is well established that its expression is elevated in respiratory syncytial virus-infected airway epithelial cells and plays a non-redundant role on anti-viral CD8 T cell responses^37^. In addition, it has been shown that epithelial cells exposed to HIV virus triggers DC-mediated mucosal infection of CD4 + T cells^38^. Therefore, if our findings will be extended in large number of COVID-19 patients around the world, they may provide evidence for TSLP beneficial role in antiviral immunity opening a window for therapeutic intervention, as human anti-TSLP antibody (Tezepelumab) have been proven effective for blocking the interaction between TSLP in asthmatic patients^39^.

In the present study, we found a correlation of adiponectin and IL-6 concentrations which could represent an additional attempt by adipose tissue to counteract inflammation mediated by the increase in IL-6. In addition, the correlation between adiponectin and ceramides confirmed the involvement of adiponectin in lipid metabolism^40^. These finding are suggestive of an active role of adipose tissue in COVID-19 onset.

Our data reinforce previous results that lipid metabolism play an important role in the viral infection cycle. Further work is necessary to investigate the role of lipids in the signaling pathway of the viral life cycle. These studies will establish new drug targets as well as the use of existing drugs.

## Limitations of the study

Our results were based on a single Italian cohort of COVID-19 patients in Campania Region; future studies in different cohorts and in healthy controls will be needed for extending the understanding of lipid dysregulation and cytokine production in COVID-19 pathogenesis. The median age of severe patients is older than non-severe, so we did not check the influence of age on our data. However, other authors did not observe impact of age and sex on omics data in COVID-19 patients^24^. Severe patients also exhibit comorbidities like obesity which may influence the lipidomic profile. Lipidomic analysis was carried out in serum but not in lung tissues or in the BALF (bronchoalveolar lavage fluid) or sputum of COVID-19 and therefore we cannot establish a direct association between lipidomic and cytokine changes with SARS-CoV-2 infection in the lung. Furthermore, only a small number of cytokines has been measured and in particular, other alarmins beside TSLP, such as IL-25 and IL-33, have not been measured.

## Materials and methods

### Patients

A Regione-Campania-cohort of 53 patients with a diagnosis of COVID-19 (SARS-CoV-2 infection) admitted at one of the following hospitals was enrolled: Department of Clinical Medicine and Surgery—Section of Infectious Diseases, University Hospital Federico II, Naples; Department of Infectious Disease and Infectious Urgencies—Division of Respiratory Infectious Disease, Cotugno Hospital, AORN dei Colli, Naples, Pathophysiology and respiratory rehabilitation-1 utsir COVID. 53 patients were enrolled (37/53 males, 70% and 16/53 females, 30%), with median age of 58 years. Patients were classified using an seven-point ordinal scale which consists of following numeric scale and scale descriptors^26^: 1, not hospitalized with resumption of normal activities; 2, not hospitalized, but unable to resume normal activities; 3, hospitalized, not requiring supplemental oxygen; 4, hospitalized, requiring supplemental oxygen; 5, hospitalized, requiring nasal high-flow oxygen therapy, non-invasive mechanical ventilation, or both; 6, hospitalized, requiring ECMO, invasive mechanical ventilation, or both; and 7, death. 1–3 were subclassified as mild patients (n = 20), 4 was subclassified as moderate patients (n = 16), and 5–7 were subclassified as severe patients (n = 17). The aliquots of serum sample were collected at hospital admission and stored at − 80 °C for subsequent analysis. The study protocol was approved by Ethics Committee at The University of Naples Federico II n°191/20. All methods and experimental procedure were performed in accordance with the relevant guidelines and regulations included in n°191/20 protocol approved by Ethics Committee at The University of Naples Federico II. The present research was performed in accordance with the Declaration of Helsinki. The present research did not involve human participants under the age of 18 years. Each patient (and/or legal guardian) gave a fully informed consent to the participation to the study and to the use of their biological samples for research purpose.

### Lipidomic analysis

Serum lipidome content was investigated by mass spectrometry-based targeted metabolomics^41,42^. The lipidomic platform was set to detect 483 lipids including 22 cholesterol esters, 34 glycosylceramides, 44 diacylglycerols, 90 glycerophospholipids, 15 sphingolipids, 242 triacylglycerols, 28 ceramides, 8 dihydroceramides according to Mxp Quant 500 protocols (Biocrates Life Sciences Innsbruck, Austria). For each analyzed sample, 10 µL of patient serum were processed and three technical replicates were carried out. Multiple reaction monitoring (MRM) was used to target and quantitate the molecular species from the different lipid classes by direct flow injection analysis (FIA), on a Triple Quad 5500 + System QTrap-Ready (AB Sciex) coupled with an Agilent 1260 Infinity II HPLC^43–46^. Briefly, 10 µL of each serum sample were added onto the wells of the 96-well extraction plate, with the exception of blank positions, PBS, calibrants, and quality controls (QC), and dried under nitrogen flow for 30 min. The samples were derivatized with 50 μL of 5% phenyl isothiocyanate at room temperature and then dried under N_2_ flow for 1 h. Metabolites were extracted with 300 µL of 5 mM ammonium acetate in methanol, shaked for 30 min (450 rpm) and eluted by centrifugation. Then, 10 µL of extract were diluted with 490 µl of FIA solvent. The injection volume into the system was 20 µL within the mobile phase (FIA solvent) at an initial flow rate of 0.03 mL/min until 1.6 min, followed by flow rates of 0.20 mL/min for 1.6 min and 0.02 mL/min for 0.20 min. The auto sampler was cooled at 10 °C. The ESI source operated in positive ion mode using the following parameters: spray voltage 55 kV, temperature of 450 °C, GS1 20, GS2 40, CUR 30, CAD 8. Data were acquired using Analyst software (version 1.7.1 Ab Sciex) and data analysis was performed used MetIDQ Oxygen 2976 (Biocrates Life Sciences Innsbruck, Austria)^47,48^.

### Lipidomic feature selection

Serum concentrations of 483 lipids from COVID-19 patients were processed by multivariate analysis using MetaboAnalyst 4.0 (http://www.metaboanalyst.ca)^49–51^. The lipidic dataset was imputed to remove the missing values, log(2)-transformed and scaled according to the Pareto scaling method. The resulted dataset was used to define PLS-DA (Partial Least Squares—Discriminant Analysis), VIP (Variable Importance in Projection) score and heatmap. Finally, univariate statistics was carried out using GraphPad Prism 8.0, and the results presented as the mean standard error of the mean (SEM). The statistical significance of the difference in serum lipid concentrations was evaluated by parametric (unpaired t-test) or non-parametric (Mann–Whitney test) tests when data failed the Shapiro–Wilk normality test. Statistical significantly changed lipids were selected using criteria of *p*-value (*p* < 0.05) and log2 fold change (FC) larger than ± 0.58, corresponding to FC (defined as ratio between lipid non-null concentrations) value of 1.5.

### Cytokine measurement

Concentrations of TSLP protein in blood serum were evaluated by enzyme-linked immunosorbent assay (ELISA) (Human TSLP Uncoated ELISA Kit; ThermoFisher) according to the manufacturer's instructions). Duplicate tests of each independent sample were conducted. 100 μL of serum samples was used for experiments. The results were expressed as protein concentration per mL of serum. Data of serum IL-17A, IL-17RA, IL-6 and TNF-α were already available from a previous study carried out on the same serum samples^26^. Serum IL-26 was measured by ELISA kit from Sigma-Aldrich (St. Louis, MO 63103 USA), in accordance with the manufacturer's instructions. The assay was performed using anti-human IL-26 precoated 96-well strip plates. Serum samples were analyzed using 100 μL of twofold diluted sample. All samples and standard solutions for calibration curves were analyzed in duplicate and mean concentrations were expressed as pg/mL.

### Adiponectin measurement

The serum adiponectin concentrations were measured by enzyme-linked immunosorbent assay (ELISA) as previously reported^21^. Calibration curve was performed and quantified using human recombinant Adiponectin (Biovendor R&D, Brno, Czech Republic) as a standard. Each sample, diluted 1:5000, was assayed three times in duplicate. In addition, adiponectin profile was analyzed by western blot analysis as previously described^21^. The western-blot filter was scanned by using Chemidoc MP imaging system (BIORAD) able to provide automatic recognition adjustment of image scanning parameters.

### Statistics

To calculate the statistical significance of changes in each cytokine in mild, moderate and severe patients a one-way ANOVA with Tukey’s multiple-comparison test was performed. To measure the strength of association between the 483 lipids and TNF-α, IL-6, IL-17A, IL-17RA, IL-26, TSLP and Adiponectin, the Spearman's rho method was used, where the value r = 1 means a perfect positive correlation and the value r = − 1 means a perfect negative correlation. The correlation was considered significant at the 0.01 level (2-tailed).

**Table 1:** Differentially abundant lipids in the severe condition versus mild condition.

Lipid	FC	log2 (FC)	*p value*	− Log (*p value*)
DG(17:0_17:1)	2.14	1.10	0.0004	3.45
TG(16:1_38:3)	1.89	0.92	0.0010	2.98
TG(20:3_34:3)	0.50	− 1.00	0.0031	2.51
Cer(d18:0/20:0)	1.55	0.63	0.0039	2.41
Cer(d18:1/23:0)	3.44	1.78	0.0042	2.37
Cer(d18:1/18:0)	1.65	0.72	0.0068	2.17
Cer(d18:1/26:1)	1.73	0.79	0.0070	2.15
PC ae C38:0	2.16	1.11	0.0100	2.00
PC ae C36:4	2.53	1.34	0.0116	1.93
TG(16:0_40:8)	1.62	0.70	0.0145	1.84
TG(17:1_36:5)	0.58	− 0.78	0.0151	1.82
TG(22:0_32:4)	1.88	0.91	0.0155	1.81
TG(18:2_36:5)	1.55	0.64	0.0230	1.64
TG(18:0_36:3)	0.59	− 0.75	0.0242	1.62
TG(18:1_35:3)	0.53	− 0.93	0.0261	1.58
TG(18:0_36:4)	0.62	− 0.69	0.0265	1.58
HexCer(d16:1/22:0)	0.61	− 0.72	0.0269	1.57
DG(16:0_20:4)	0.57	− 0.81	0.0270	1.57
TG(16:0_33:1)	0.62	− 0.70	0.0304	1.52
TG(18:1_34:3)	1.51	0.59	0.0314	1.50
SM C24:0	2.25	1.17	0.0318	1.50
TG(16:0_34:0)	0.60	− 0.74	0.0335	1.48
TG(20:2_32:1)	0.58	− 0.79	0.0362	1.44
TG(18:2_33:2)	1.54	0.62	0.0414	1.38
DG(18:1_20:2)	2.30	1.20	0.0416	1.38
Hex2Cer(d18:1/24:0)	0.66	− 0.61	0.0476	1.32
Cer(d18:0/26:1OH)	1.96	0.97	0.0488	1.31
TG(17:2_36:4)	0.64	− 0.64	0.0493	1.31

**Table 2:** Differentially abundant lipids in the severe condition versus medium condition.

Lipid	FC	log2 (FC)	*p value*	− Log (*p value*)
DG(17:0_17:1)	2.51	1.32	0.0017	2.78
TG(20:4_36:3)	2.00	1.00	0.0024	2.61
TG(18:1_32:1)	0.54	− 0.90	0.0094	2.03
TG(20:4_32:0)	0.55	− 0.86	0.0115	1.94
TG(18:1_38:7)	2.11	1.08	0.0140	1.85
TG(22:4_34:2)	0.55	− 0.86	0.0165	1.78
TG(18:2_34:2)	2.05	1.04	0.0176	1.75
CE17:1	1.56	0.64	0.0185	1.73
TG(20:3_36:4)	1.70	0.76	0.0216	1.67
CE20:1	0.51	− 0.97	0.0226	1.65
TG(20:4_34:0)	0.58	− 0.79	0.0239	1.62
TG(17:0_32:1)	0.61	− 0.72	0.0263	1.58
Cer(d18:0/26:1OH)	2.18	1.12	0.0266	1.57
PC ae C44:5	0.60	− 0.73	0.0305	1.52
TG(18:3_36:2)	1.55	0.63	0.0384	1.42
lysoPC a C16:0	2.32	1.22	0.0421	1.38
HexCer(d18:1/22:0)	0.57	− 0.81	0.0493	1.31
TG(18:0_32:1)	0.65	− 0.63	0.0496	1.30

**Table 3:** Adiponectin serum concentrations (µg/ml) in COVID− 19 patients.

Patients	T0	T1	*p-value*
Covid-19 patients mild	17.2 ± 3.14	15.6 ± 1.38	ns
Covid-19 patients moderate	19.3 ± 4.92	19.0 ± 0.57	ns
Covid-19 patients severe	21.4 ± 2.67	19.8 ± 2.57	ns

## Supplementary Information

Supplementary Information 1.

Supplementary Information 2.

Supplementary Information 3.

Supplementary Information 4.

Supplementary Information 5.
