# Supplementary material for: Dysregulation of lipid metabolism and pathological inflammation in patients with COVID-19
Source: Sci Rep. 2021 Feb 3;11:2941. doi: 10.1038/s41598-021-82426-7 (PMC7859398; doi:10.1038/s41598-021-82426-7)
Supplement: Supplementary file 1 — Supplementary Information 1. [file 41598_2021_82426_MOESM1_ESM.pdf]

Dysregulation of lipid metabolism and pathological inflammation in patients with COVID-19

Marianna Caterino[1,2°], Monica Gelzo[1,2°], Stefano Sol[1,3°], Roberta Fedele[1], Anna Annunziata[4], Cecilia Calabrese[5], Giuseppe Fiorentino[4], Maurizio D'Abbraccio[6], Chiara Dell'Isola[6], Francesco Maria Fusco[6], Roberto Parrella[6], Gabriella Fabbrocini[7], Ivan Gentile[7], Immacolata Andolfo[1,2], Mario Capasso[1,2], Michele Costanzo[1,2], Aurora Daniele[1,8], Emanuela Marchese[1,9], Rita Polito[1,10], Roberta Russo[1,2], Caterina Missero[1,3]*, Margherita Ruoppolo[1,2],* and Giuseppe Castaldo[1,2]*

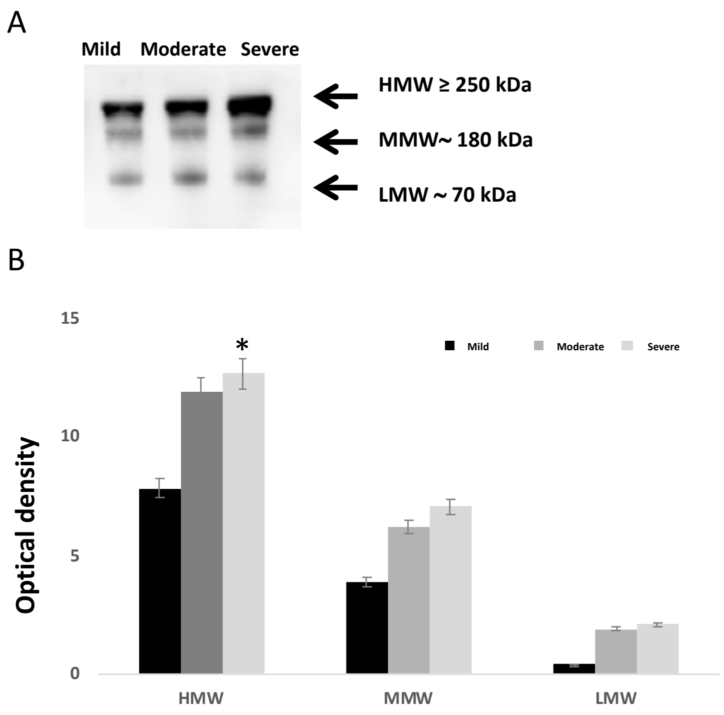

Supplementary Figure 1

Supplementary Figure 1. Serum Adiponectin oligomers in COVID-19 patient serum (A) Representative blot image of adiponectin different oligomers (HMW, MMW, LMW) in serum of severe, moderate and mild Covid-19 patients. (B) Graphical representation of pixel quantization of all analysed patients.

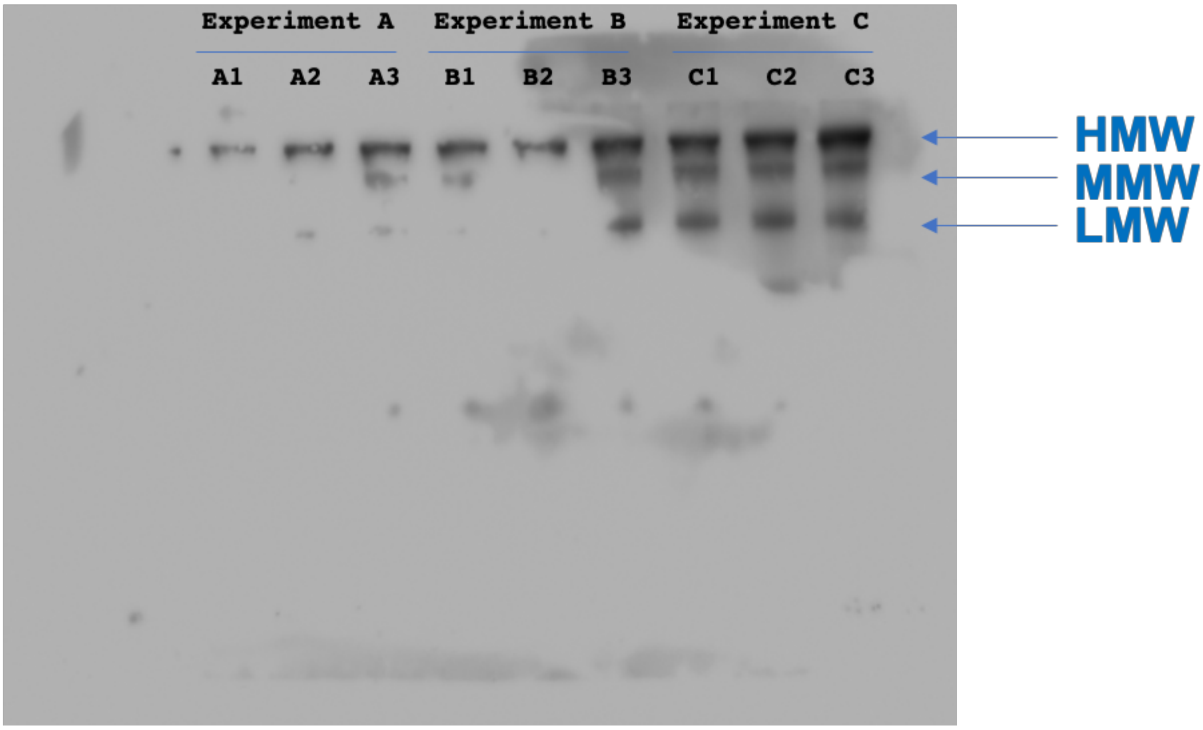

Supplementary Figure 2

Supplementary Figure 2. Serum Adiponectin oligomers in COVID-19 patient serum. Full-lenght image of western-blot of the adiponectin different oligomers (HMW, MMW, LMW) in three Covid-19 patient subgroups (A, B, C), including mild (A1, B1 and C1), moderate (A2, B2 and C2) and severe (A3, B3 and C3) serum patients. The western-blot filter was scanned by using Chemidoc MP imaging system (BIORAD) able to provide automatic recognition adjustment of image scanning parameters. The subgroup C was used as representative blot image of adiponectin in Supplementary Figure 1S.
