# Supplementary material for: Dysregulation of lipid metabolism and pathological inflammation in patients with COVID-19
Source: Sci Rep. 2021 Feb 3;11:2941. doi: 10.1038/s41598-021-82426-7 (PMC7859398; doi:10.1038/s41598-021-82426-7)
Supplement: Supplementary file 2 — Supplementary Information 2. [file 41598_2021_82426_MOESM2_ESM.pdf]

Dysregulation of lipid metabolism and pathological inflammation in patients with COVID-19

Marianna Caterino[1,2°], Monica Gelzo[1,2°], Stefano Sol[1,3°], Roberta Fedele[1], Anna Annunziata[4], Cecilia Calabrese[5], Giuseppe Fiorentino[4], Maurizio D'Abbraccio[6], Chiara Dell'Isola[6], Francesco Maria Fusco[6], Roberto Parrella[6], Gabriella Fabbrocini[7], Ivan Gentile[7], Immacolata Andolfo[1,2], Mario Capasso[1,2], Michele Costanzo[1,2], Aurora Daniele[1,8], Emanuela Marchese[1,9], Rita Polito[1,10], Roberta Russo[1,2], Caterina Missero[1,3*], Margherita Ruoppolo[1,2,*] and Giuseppe Castaldo[1,2*]

Table 1S. Lipid descriptions

Metabolite	Biochemical Name	Class
CE(14:0)	Cholesterol ester (14:0)	cholesterol ester
CE(14:1)	Cholesterol ester (14:1)	cholesterol ester
CE(15:0)	Cholesterol ester (15:0)	cholesterol ester
CE(15:1)	Cholesterol ester (15:1)	cholesterol ester
CE(16:0)	Cholesterol ester (16:0)	cholesterol ester
CE(16:1)	Cholesterol ester (16:1)	cholesterol ester
CE(17:0)	Cholesterol ester (17:0)	cholesterol ester
CE(17:1)	Cholesterol ester (17:1)	cholesterol ester
CE(18:0)	Cholesterol ester (18:0)	cholesterol ester
CE(18:1)	Cholesterol ester (18:1)	cholesterol ester
CE(18:2)	Cholesterol ester (18:2)	cholesterol ester
CE(18:3)	Cholesterol ester (18:3)	cholesterol ester
CE(20:0)	Cholesterol ester (20:0)	cholesterol ester
CE(20:1)	Cholesterol ester (20:1)	cholesterol ester
CE(20:3)	Cholesterol ester (20:3)	cholesterol ester
CE(20:4)	Cholesterol ester (20:4)	cholesterol ester
CE(20:5)	Cholesterol ester (20:5)	cholesterol ester
CE(22:0)	Cholesterol ester (22:0)	cholesterol ester
CE(22:1)	Cholesterol ester (22:1)	cholesterol ester
CE(22:2)	Cholesterol ester (22:2)	cholesterol ester
CE(22:5)	Cholesterol ester (22:5)	cholesterol ester
CE(22:6)	Cholesterol ester (22:6)	cholesterol ester
Hex-Cer(d16:1/22:0)	Hexosylceramide (d16:1/22:0)	glycosylceramides
Hex- Cer(d16:1/24:0)	Hexosylceramide (d16:1/24:0)	glycosylceramides
Hex- Cer(d18:1/14:0)	Hexosylceramide (d18:1/14:0)	glycosylceramides
Hex- Cer(d18:1/16:0)	Hexosylceramide (d18:1/16:0)	glycosylceramides
Hex-Cer(d18:1/18:0)	Hexosylceramide (d18:1/18:0)	glycosylceramides
Hex-Cer(d18:1/18:1)	Hexosylceramide (d18:1/18:1)	glycosylceramides
Hex- Cer(d18:1/20:0)	Hexosylceramide (d18:1/20:0)	glycosylceramides
Hex- Cer(d18:1/22:0)	Hexosylceramide (d18:1/22:0)	glycosylceramides
Hex- Cer(d18:1/23:0)	Hexosylceramide (d18:1/23:0)	glycosylceramides
Hex- Cer(d18:1/24:0)	Hexosylceramide (d18:1/24:0)	glycosylceramides
Hex-Cer(d18:1/24:1)	Hexosylceramide (d18:1/24:1)	glycosylceramides
Hex-Cer(d18:1/26:0)	Hexosylceramide (d18:1/26:0)	glycosylceramides
Hex- Cer(d18:1/26:1)	Hexosylceramide (d18:1/26:1)	glycosylceramides
Hex- Cer(d18:2/16:0)	Hexosylceramide (d18:2/16:0)	glycosylceramides
Hex- Cer(d18:2/18:0)	Hexosylceramide (d18:2/18:0)	glycosylceramides
Hex- Cer(d18:2/20:0)	Hexosylceramide (d18:2/20:0)	glycosylceramides
Hex-Cer(d18:2/22:0)	Hexosylceramide (d18:2/22:0)	glycosylceramides
Hex-Cer(d18:2/23:0)	Hexosylceramide (d18:2/23:0)	glycosylceramides
Hex-Cer(d18:2/24:0)	Hexosylceramide (d18:2/24:0)	glycosylceramides

Hex2Cer(d18:1/ 14:0)	Dihexosylceramide (d18:1/14:0)	glycosylceramides
Hex2Cer(d18:1/ 16:0)	Dihexosylceramide (d18:1/16:0)	glycosylceramides
Hex2Cer(d18:1/ 18:0)	Dihexosylceramide (d18:1/18:0)	glycosylceramides
Hex2Cer(d18:1/20:0)	Dihexosylceramide(d18:1/20:0)	glycosylceramides
Hex2Cer(d18:1/22:0)	Dihexosylceramide(d18:1/22:0)	glycosylceramides
Hex2Cer(d18:1/ 24:0)	Dihexosylceramide (d18:1/24:0)	glycosylceramides
Hex2Cer(d18:1/ 24:1)	Dihexosylceramide (d18:1/24:1)	glycosylceramides
Hex2Cer(d18:1/ 26:0)	Dihexosylceramide (d18:1/26:0)	glycosylceramides
Hex2Cer(d18:1/26:1)	Dihexosylceramide(d18:1/26:1)	glycosylceramides
Hex3Cer(d18:1/16:0)	Trihexosylceramide(d18:1/16:0)	glycosylceramides
Hex3Cer(d18:1/18:0)	Trihexosylceramide(d18:1/18:0)	glycosylceramides
Hex3Cer(d18:1/ 24:1)	Trihexosylceramide (d18:1/24:1)	glycosylceramides
Hex3Cer(d18:1/ 26:1)	Trihexosylceramide (d18:1/26:1)	glycosylceramides
Hex3Cer(d18:1_20:0)	Trihexosylceramide (d18:1_20:0)	glycosylceramides
Hex3Cer(d18:1_22:0)	Trihexosylceramide (d18:1_22:0)	glycosylceramides
DG(14:0_14:0)	Diacylglyceride (14:0_14:0)	diacylglycerols
DG(14:0_18:1)	Diacylglyceride (14:0_18:1)	diacylglycerols
DG(14:0_18:2)	Diacylglyceride (14:0_18:2)	diacylglycerols
DG(14:0_20:0)	Diacylglyceride (14:0_20:0)	diacylglycerols
DG(14:1_18:1)	Diacylglyceride (14:1_18:1)	diacylglycerols
DG(14:1_20:2)	Diacylglyceride (14:1_20:2)	diacylglycerols
DG(16:0_16:0)	Diacylglyceride (16:0_16:0)	diacylglycerols
DG(16:0_16:1)	Diacylglyceride (16:0_16:1)	diacylglycerols
DG(16:0_18:1)	Diacylglyceride (16:0_18:1)	diacylglycerols
DG(16:0_18:2)	Diacylglyceride (16:0_18:2)	diacylglycerols
DG(16:0_20:0)	Diacylglyceride (16:0_20:0)	diacylglycerols
DG(16:0_20:3)	Diacylglyceride (16:0_20:3)	diacylglycerols
DG(16:0_20:4)	Diacylglyceride (16:0_20:4)	diacylglycerols
DG(16:1_18:0)	Diacylglyceride (16:1_18:0)	diacylglycerols
DG(16:1_18:1)	Diacylglyceride (16:1_18:1)	diacylglycerols
DG(16:1_18:2)	Diacylglyceride (16:1_18:2)	diacylglycerols
DG(16:1_20:0)	Diacylglyceride (16:1_20:0)	diacylglycerols
DG(17:0_17:1)	Diacylglyceride (17:0_17:1)	diacylglycerols
DG(17:0_18:1)	Diacylglyceride (17:0_18:1)	diacylglycerols
DG(18:0_20:0)	Diacylglyceride (18:0_20:0)	diacylglycerols
DG(18:0_20:4)	Diacylglyceride (18:0_20:4)	diacylglycerols
DG(18:1_18:1)	Diacylglyceride (18:1_18:1)	diacylglycerols
DG(18:1_18:2)	Diacylglyceride (18:1_18:2)	diacylglycerols
DG(18:1_18:3)	Diacylglyceride (18:1_18:3)	diacylglycerols
DG(18:1_18:4)	Diacylglyceride (18:1_18:4)	diacylglycerols
DG(18:1_20:0)	Diacylglyceride (18:1_20:0)	diacylglycerols
DG(18:1_20:1)	Diacylglyceride (18:1_20:1)	diacylglycerols
DG(18:1_20:2)	Diacylglyceride (18:1_20:2)	diacylglycerols
DG(18:1_20:3)	Diacylglyceride (18:1_20:3)	diacylglycerols
DG(18:1_20:4)	Diacylglyceride (18:1_20:4)	diacylglycerols
DG(18:1_22:5)	Diacylglyceride (18:1_22:5)	diacylglycerols
DG(18:1_22:6)	Diacylglyceride (18:1_22:6)	diacylglycerols

DG(18:2_18:2)	Diacylglyceride (18:2_18:2)	diacylglycerols
DG(18:2_18:3)	Diacylglyceride (18:2_18:3)	diacylglycerols
DG(18:2_18:4)	Diacylglyceride (18:2_18:4)	diacylglycerols
DG(18:2_20:0)	Diacylglyceride(18:2_20:0)	diacylglycerols
DG(18:2_20:4)	Diacylglyceride (18:2_20:4)	diacylglycerols
DG(18:3_18:3)	Diacylglyceride(18:3_18:3)	diacylglycerols
DG(18:3_20:2)	Diacylglyceride (18:3_20:2)	diacylglycerols
DG(21:0_22:6)	Diacylglyceride (21:0_22:6)	diacylglycerols
DG(22:1_22:2)	Diacylglyceride (22:1_22:2)	diacylglycerols
DG- O(14:0_18:2)	Alkylacylglycerol (O- 18:2_14:0)	diacylglycerols
DG-O(16:0_18:1)	Alkylacylglycerol (O-18:1_16:0)	diacylglycerols
DG-O(16:0_20:4)	Alkylacylglycerol (O-18:2_18:2)	diacylglycerols
lysoPC a C14:0	lysoPhosphatidyl- choline acyl C14:0	glycerophospholipids
lysoPC a C16:0	lysoPhosphatidyl- choline acyl C16:0	glycerophospholipids
lysoPC a C16:1	lysoPhosphatidyl- choline acyl C16:1	glycerophospholipids
lysoPC a C17:0	lysoPhosphatidyl-choline acyl C17:0	glycerophospholipids
lysoPC a C18:0	lysoPhosphatidyl-choline acyl C18:0	glycerophospholipids
lysoPC a C18:1	lysoPhosphatidyl-choline acyl C18:1	glycerophospholipids
lysoPC a C18:2	lysoPhosphatidyl- choline acyl C18:2	glycerophospholipids
lysoPC a C20:3	lysoPhosphatidyl- choline acyl C20:3	glycerophospholipids
lysoPC a C20:4	lysoPhosphatidyl- choline acyl C20:4	glycerophospholipids
lysoPC a C24:0	lysoPhosphatidyl- choline acyl C24:0	glycerophospholipids
lysoPC a C26:0	lysoPhosphatidyl-choline acyl C26:0	glycerophospholipids
lysoPC a C26:1	lysoPhosphatidyl-choline acyl C26:1	glycerophospholipids
lysoPC a C28:0	lysoPhosphatidyl-choline acyl C28:0	glycerophospholipids
lysoPC a C28:1	lysoPhosphatidyl- choline acyl C28:1	glycerophospholipids
PC aa C24:0	Phosphatidylcholine diacyl C24:0	glycerophospholipids
PC aa C26:0	Phosphatidylcholine diacyl C26:0	glycerophospholipids
PC aa C28:1	Phosphatidylcholine diacyl C28:1	glycerophospholipids
PC aa C30:0	Phosphatidylcholine diacyl C30:0	glycerophospholipids
PC aa C30:2	Phosphatidylcholine diacyl C30:2	glycerophospholipids
PC aa C32:0	Phosphatidylcholine diacyl C32:0	glycerophospholipids
PC aa C32:1	Phosphatidylcholine diacyl C32:1	glycerophospholipids
PC aa C32:2	Phosphatidylcholine diacyl C32:2	glycerophospholipids
PC aa C32:3	Phosphatidylcholine diacyl C32:3	glycerophospholipids
PC aa C34:1	Phosphatidylcholine diacyl C34:1	glycerophospholipids
PC aa C34:2	Phosphatidylcholine diacyl C34:2	glycerophospholipids
PC aa C34:3	Phosphatidylcholine diacyl C34:3	glycerophospholipids
PC aa C34:4	Phosphatidylcholine diacyl C34:4	glycerophospholipids
PC aa C36:0	Phosphatidylcholine diacyl C36:0	glycerophospholipids
PC aa C36:1	Phosphatidylcholine diacyl C36:1	glycerophospholipids
PC aa C36:2	Phosphatidylcholine diacyl C36:2	glycerophospholipids
PC aa C36:3	Phosphatidylcholine diacyl C36:3	glycerophospholipids
PC aa C36:4	Phosphatidylcholine diacyl C36:4	glycerophospholipids
PC aa C36:5	Phosphatidylcholine diacyl C36:5	glycerophospholipids
PC aa C36:6	Phosphatidylcholine diacyl C36:6	glycerophospholipids
PC aa C38:0	Phosphatidylcholine diacyl C38:0	glycerophospholipids

PC aa C38:1	Phosphatidylcholine diacyl C38:1	glycerophospholipids
PC aa C38:3	Phosphatidylcholine diacyl C38:3	glycerophospholipids
PC aa C38:4	Phosphatidylcholine diacyl C38:4	glycerophospholipids
PC aa C38:5	Phosphatidylcholine diacyl C38:5	glycerophospholipids
PC aa C38:6	Phosphatidylcholine diacyl C38:6	glycerophospholipids
PC aa C40:1	Phosphatidylcholine diacyl C40:1	glycerophospholipids
PC aa C40:2	Phosphatidylcholine diacyl C40:2	glycerophospholipids
PC aa C40:3	Phosphatidylcholine diacyl C40:3	glycerophospholipids
PC aa C40:4	Phosphatidylcholine diacyl C40:4	glycerophospholipids
PC aa C40:5	Phosphatidylcholine diacyl C40:5	glycerophospholipids
PC aa C40:6	Phosphatidylcholine diacyl C40:6	glycerophospholipids
PC aa C42:0	Phosphatidylcholine diacyl C42:0	glycerophospholipids
PC aa C42:1	Phosphatidylcholine diacyl C42:1	glycerophospholipids
PC aa C42:2	Phosphatidylcholine diacyl C42:2	glycerophospholipids
PC aa C42:4	Phosphatidylcholine diacyl C42:4	glycerophospholipids
PC aa C42:5	Phosphatidylcholine diacyl C42:5	glycerophospholipids
PC aa C42:6	Phosphatidylcholine diacyl C42:6	glycerophospholipids
PC ae C30:0	Phosphatidylcholine acyl-alkyl C30:0	glycerophospholipids
PC ae C30:1	Phosphatidylcholine acyl-alkyl C30:1	glycerophospholipids
PC ae C30:2	Phosphatidylcholine acyl-alkyl C30:2	glycerophospholipids
PC ae C32:1	Phosphatidylcholine acyl-alkyl C32:1	glycerophospholipids
PC ae C32:2	Phosphatidylcholine acyl-alkyl C32:2	glycerophospholipids
PC ae C34:0	Phosphatidylcholine acyl-alkyl C34:0	glycerophospholipids
PC ae C34:1	Phosphatidylcholine acyl-alkyl C34:1	glycerophospholipids
PC ae C34:2	Phosphatidylcholine acyl-alkyl C34:2	glycerophospholipids
PC ae C34:3	Phosphatidylcholine acyl-alkyl C34:3	glycerophospholipids
PC ae C36:0	Phosphatidylcholine acyl-alkyl C36:0	glycerophospholipids
PC ae C36:1	Phosphatidylcholine acyl-alkyl C36:1	glycerophospholipids
PC ae C36:2	Phosphatidylcholine acyl-alkyl C36:2	glycerophospholipids
PC ae C36:3	Phosphatidylcholine acyl-alkyl C36:3	glycerophospholipids
PC ae C36:4	Phosphatidylcholine acyl-alkyl C36:4	glycerophospholipids
PC ae C36:5	Phosphatidylcholine acyl-alkyl C36:5	glycerophospholipids
PC ae C38:0	Phosphatidylcholine acyl-alkyl C38:0	glycerophospholipids
PC ae C38:1	Phosphatidylcholine acyl-alkyl C38:1	glycerophospholipids
PC ae C38:2	Phosphatidylcholine acyl-alkyl C38:2	glycerophospholipids
PC ae C38:3	Phosphatidylcholine acyl-alkyl C38:3	glycerophospholipids
PC ae C38:4	Phosphatidylcholine acyl-alkyl C38:4	glycerophospholipids
PC ae C38:5	Phosphatidylcholine acyl-alkyl C38:5	glycerophospholipids
PC ae C38:6	Phosphatidylcholine acyl-alkyl C38:6	glycerophospholipids
PC ae C40:1	Phosphatidylcholine acyl-alkyl C40:1	glycerophospholipids
PC ae C40:2	Phosphatidylcholine acyl-alkyl C40:2	glycerophospholipids
PC ae C40:3	Phosphatidylcholine acyl-alkyl C40:3	glycerophospholipids
PC ae C40:4	Phosphatidylcholine acyl-alkyl C40:4	glycerophospholipids
PC ae C40:5	Phosphatidylcholine acyl-alkyl C40:5	glycerophospholipids
PC ae C40:6	Phosphatidylcholine acyl-alkyl C40:6	glycerophospholipids
PC ae C42:0	Phosphatidylcholine acyl-alkyl C42:0	glycerophospholipids
PC ae C42:1	Phosphatidylcholine acyl-alkyl C42:1	glycerophospholipids

PC ae C42:2	Phosphatidylcholine acyl-alkyl C42:2	glycerophospholipids
PC ae C42:3	Phosphatidylcholine acyl-alkyl C42:3	glycerophospholipids
PC ae C42:4	Phosphatidylcholine acyl-alkyl C42:4	glycerophospholipids
PC ae C42:5	Phosphatidylcholine acyl-alkyl C42:5	glycerophospholipids
PC ae C44:3	Phosphatidylcholine acyl-alkyl C44:3	glycerophospholipids
PC ae C44:4	Phosphatidylcholine acyl-alkyl C44:4	glycerophospholipids
PC ae C44:5	Phosphatidylcholine acyl-alkyl C44:5	glycerophospholipids
PC ae C44:6	Phosphatidylcholine acyl-alkyl C44:6	glycerophospholipids
SM (OH) C14:1	Hydroxysphingomy- eline C14:1	sphingolipids
SM (OH) C16:1	Sphingomyeline C16:0	sphingolipids
SM (OH) C22:1	Sphingomyeline C16:1	sphingolipids
SM (OH) C22:2	Hydroxysphingomy-eline C16:1	sphingolipids
SM (OH) C24:1	Sphingomyeline C18:0	sphingolipids
SM C16:0	Sphingomyeline C18:1	sphingolipids
SM C16:1	Sphingomyeline C20:2	sphingolipids
SM C18:0	Sphingomyeline C22:3	sphingolipids
SM C18:1	Hydroxysphingomy- eline C22:1	sphingolipids
SM C20:2	Hydroxysphingomy- eline C22:2	sphingolipids
SM C22:3	Sphingomyeline C24:0	sphingolipids
SM C24:0	Sphingomyeline C24:1	sphingolipids
SM C24:1	Hydroxysphingomy-eline C24:1	sphingolipids
SM C26:0	Sphingomyeline C26:0	sphingolipids
SM C26:1	Sphingomyeline C26:1	sphingolipids
TG(14:0_32:2)	Triacylglceride (46:2) w/ FA (14:0)	triacylglycerols
TG(14:0_34:0)	Triacylglceride (48:0) w/ FA (14:0)	triacylglycerols
TG(14:0_34:1)	Triacylglceride (48:1) w/ FA (14:0)	triacylglycerols
TG(14:0_34:2)	Triacylglceride (48:2) w/ FA (14:0)	triacylglycerols
TG(14:0_34:3)	Triacylglceride (48:3) w/ FA (14:0)	triacylglycerols
TG(14:0_35:1)	Triacylglceride (49:1) w/ FA (14:0)	triacylglycerols
TG(14:0_35:2)	Triacylglceride (49:2) w/ FA (14:0)	triacylglycerols
TG(14:0_36:1)	Triacylglceride (50:1) w/ FA (14:0)	triacylglycerols
TG(14:0_36:2)	Triacylglceride (50:2) w/ FA (14:0)	triacylglycerols
TG(14:0_36:3)	Triacylglceride (50:3) w/ FA (14:0)	triacylglycerols
TG(14:0_36:4)	Triacylglceride (50:4) w/ FA (14:0)	triacylglycerols
TG(14:0_38:4)	Triacylglceride (52:4) w/ FA (14:0)	triacylglycerols
TG(14:0_38:5)	Triacylglceride (52:5) w/ FA (14:0)	triacylglycerols
TG(14:0_39:3)	Triacylglceride (53:3) w/ FA (14:0)	triacylglycerols
TG(16:0_28:1)	Triacylglceride (44:1) w/ FA (16:0)	triacylglycerols
TG(16:0_28:2)	Triacylglceride (44:2) w/ FA (16:0)	triacylglycerols
TG(16:0_30:2)	Triacylglceride (46:2) w/ FA (16:0)	triacylglycerols
TG(16:0_32:0)	Triacylglceride (48:0) w/ FA (16:0)	triacylglycerols
TG(16:0_32:1)	Triacylglceride (48:1) w/ FA (16:0)	triacylglycerols
TG(16:0_32:2)	Triacylglceride (48:2) w/ FA (16:0)	triacylglycerols
TG(16:0_32:3)	Triacylglceride (48:3) w/ FA (16:0)	triacylglycerols
TG(16:0_33:1)	Triacylglceride (49:1) w/ FA (16:0)	triacylglycerols
TG(16:0_33:2)	Triacylglceride (49:2) w/ FA (16:0)	triacylglycerols
TG(16:0_34:0)	Triacylglceride (50:0) w/ FA (16:0)	triacylglycerols

TG(16:0_34:1)	Triacylglceride (50:1) w/ FA (16:0)	triacylglycerols
TG(16:0_34:2)	Triacylglceride (50:2) w/ FA (16:0)	triacylglycerols
TG(16:0_34:3)	Triacylglceride (50:3) w/ FA (16:0)	triacylglycerols
TG(16:0_34:4)	Triacylglceride (50:4) w/ FA (16:0)	triacylglycerols
TG(16:0_35:1)	Triacylglceride (51:1) w/ FA (16:0)	triacylglycerols
TG(16:0_35:2)	Triacylglceride (51:2) w/ FA (16:0)	triacylglycerols
TG(16:0_35:3)	Triacylglceride (51:3) w/ FA (16:0)	triacylglycerols
TG(16:0_36:2)	Triacylglceride (52:2) w/ FA (16:0)	triacylglycerols
TG(16:0_36:3)	Triacylglceride (52:3) w/ FA (16:0)	triacylglycerols
TG(16:0_36:4)	Triacylglceride (52:4) w/ FA (16:0)	triacylglycerols
TG(16:0_36:5)	Triacylglceride (52:5) w/ FA (16:0)	triacylglycerols
TG(16:0_36:6)	Triacylglceride (52:6) w/ FA (16:0)	triacylglycerols
TG(16:0_37:3)	Triacylglceride (53:3) w/ FA (16:0)	triacylglycerols
TG(16:0_38:1)	Triacylglceride (54:1) w/ FA (16:0	triacylglycerols
TG(16:0_38:2)	Triacylglceride (54:2) w/ FA (16:0)	triacylglycerols
TG(16:0_38:3)	Triacylglceride (54:3) w/ FA (16:0)	triacylglycerols
TG(16:0_38:4)	Triacylglceride (54:4) w/ FA (16:0)	triacylglycerols
TG(16:0_38:5)	Triacylglceride (54:5) w/ FA (16:0)	triacylglycerols
TG(16:0_38:6)	Triacylglceride (54:6) w/ FA (16:0)	triacylglycerols
TG(16:0_38:7)	Triacylglceride (54:7) w/ FA (16:0)	triacylglycerols
TG(16:0_40:6)	Triacylglceride (56:6) w/ FA (16:0)	triacylglycerols
TG(16:0_40:7)	Triacylglceride (56:7) w/ FA (16:0)	triacylglycerols
TG(16:0_40:8)	Triacylglceride (56:8) w/ FA (16:0)	triacylglycerols
TG(16:1_28:0)	Triacylglceride (44:1) w/ FA (16:1)	triacylglycerols
TG(16:1_30:1)	Triacylglceride (46:2) w/ FA (16:1)	triacylglycerols
TG(16:1_32:0)	Triacylglceride (48:1) w/ FA (16:1)	triacylglycerols
TG(16:1_32:1)	Triacylglceride (48:2) w/ FA (16:1)	triacylglycerols
TG(16:1_32:2)	Triacylglceride (48:3) w/ FA (16:1)	triacylglycerols
TG(16:1_33:1)	Triacylglceride (49:2) w/ FA (16:1)	triacylglycerols
TG(16:1_34:0)	Triacylglceride (50:1) w/ FA (16:1)	triacylglycerols
TG(16:1_34:1)	Triacylglceride (50:2) w/ FA (16:1)	triacylglycerols
TG(16:1_34:2)	Triacylglceride (50:3) w/ FA (16:1)	triacylglycerols
TG(16:1_34:3)	Triacylglceride (50:4) w/ FA (16:1)	triacylglycerols
TG(16:1_36:1)	Triacylglceride (52:2) w/ FA (16:1)	triacylglycerols
TG(16:1_36:2)	Triacylglceride (52:3) w/ FA (16:1)	triacylglycerols
TG(16:1_36:3)	Triacylglceride (52:4) w/ FA (16:1)	triacylglycerols
TG(16:1_36:4)	Triacylglceride (52:5) w/ FA (16:1)	triacylglycerols
TG(16:1_36:5)	Triacylglceride (52:6) w/ FA (16:1)	triacylglycerols
TG(16:1_38:3)	Triacylglceride (54:4) w/ FA (16:1)	triacylglycerols
TG(16:1_38:4)	Triacylglceride (54:5) w/ FA (16:1)	triacylglycerols
TG(16:1_38:5)	Triacylglceride (54:6) w/ FA (16:1)	triacylglycerols
TG(17:0_32:1)	Triacylglceride (49:1) w/ FA (17:0)	triacylglycerols
TG(17:0_34:1)	Triacylglceride (51:1) w/ FA (17:0)	triacylglycerols
TG(17:0_34:2)	Triacylglceride (51:2) w/ FA (17:0)	triacylglycerols
TG(17:0_34:3)	Triacylglceride (51:3) w/ FA (17:0)	triacylglycerols
TG(17:0_36:3)	Triacylglceride (53:3) w/ FA (17:0)	triacylglycerols
TG(17:0_36:4)	Triacylglceride (53:4) w/ FA (17:0)	triacylglycerols

TG(17:1_32:1)	Triacylglceride (49:2) w/ FA (17:1)	triacylglycerols
TG(17:1_34:1)	Triacylglceride(51:2) w/ FA (17:1)	triacylglycerols
TG(17:1_34:2)	Triacylglceride (51:3) w/ FA (17:1)	triacylglycerols
TG(17:1_34:3)	Triacylglceride (51:4) w/ FA (17:1)	triacylglycerols
TG(17:1_36:3)	Triacylglceride (53:4) w/ FA (17:1)	triacylglycerols
TG(17:1_36:4)	Triacylglceride (53:5) w/ FA (17:1)	triacylglycerols
TG(17:1_36:5)	Triacylglceride (53:6) w/ FA (17:1)	triacylglycerols
TG(17:1_38:5)	Triacylglceride (55:6) w/ FA (17:1)	triacylglycerols
TG(17:1_38:6)	Triacylglceride (55:7) w/ FA (17:1)	triacylglycerols
TG(17:1_38:7)	Triacylglceride (55:8) w/ FA (17:1)	triacylglycerols
TG(17:2_34:2)	Triacylglceride (51:4) w/ FA (17:2)	triacylglycerols
TG(17:2_34:3)	Triacylglceride (51:5) w/ FA (17:2)	triacylglycerols
TG(17:2_36:2)	Triacylglceride (53:4) w/ FA (17:2)	triacylglycerols
TG(17:2_36:3)	Triacylglceride (53:5) w/ FA (17:2)	triacylglycerols
TG(17:2_36:4)	Triacylglceride (53:6) w/ FA (17:2)	triacylglycerols
TG(17:2_38:5)	Triacylglceride (55:7) w/ FA (17:2)	triacylglycerols
TG(17:2_38:6)	Triacylglceride (55:8) w/ FA (17:2)	triacylglycerols
TG(17:2_38:7)	Triacylglceride (55:9) w/ FA (17:2)	triacylglycerols
TG(18:0_30:0)	Triacylglceride (48:0) w/ FA (18:0)	triacylglycerols
TG(18:0_30:1)	Triacylglceride (48:1) w/ FA (18:0)	triacylglycerols
TG(18:0_32:0)	Triacylglceride(50:0) w/ FA (18:0)	triacylglycerols
TG(18:0_32:1)	Triacylglceride (50:1) w/ FA (18:0)	triacylglycerols
TG(18:0_32:2)	Triacylglceride (50:2) w/ FA (18:0)	triacylglycerols
TG(18:0_34:2)	Triacylglceride (52:2) w/ FA (18:0)	triacylglycerols
TG(18:0_34:3)	Triacylglceride (52:3) w/ FA (18:0)	triacylglycerols
TG(18:0_36:1)	Triacylglceride (54:1)w/ FA (18:0)	triacylglycerols
TG(18:0_36:2)	Triacylglceride (54:2) w/ FA (18:0)	triacylglycerols
TG(18:0_36:3)	Triacylglceride (54:3) w/ FA (18:0)	triacylglycerols
TG(18:0_36:4)	Triacylglceride (54:4) w/ FA (18:0)	triacylglycerols
TG(18:0_36:5)	Triacylglceride (54:5) w/ FA (18:0)	triacylglycerols
TG(18:0_38:6)	Triacylglceride (56:6) w/ FA (18:0)	triacylglycerols
TG(18:0_38:7)	Triacylglceride (56:7) w/ FA (18:0)	triacylglycerols
TG(18:1_26:0)	Triacylglceride (44:1) w/ FA (18:1)	triacylglycerols
TG(18:1_28:1)	Triacylglceride (46:2) w/ FA (18:1)	triacylglycerols
TG(18:1_30:0)	Triacylglceride (48:1) w/ FA (18:1)	triacylglycerols
TG(18:1_30:1)	Triacylglceride (48:2) w/ FA (18:1)	triacylglycerols
TG(18:1_30:2)	Triacylglceride (48:3) w/ FA (18:1)	triacylglycerols
TG(18:1_31:0)	Triacylglceride (49:1) w/ FA (18:1)	triacylglycerols
TG(18:1_32:0)	Triacylglceride (50:1) w/ FA (18:1)	triacylglycerols
TG(18:1_32:1)	Triacylglceride (50:2) w/ FA (18:1)	triacylglycerols
TG(18:1_32:2)	Triacylglceride (50:3) w/ FA (18:1)	triacylglycerols
TG(18:1_32:3)	Triacylglceride (50:4) w/ FA (18:1)	triacylglycerols
TG(18:1_33:0)	Triacylglceride (51:1) w/ FA (18:1)	triacylglycerols
TG(18:1_33:1)	Triacylglceride (51:2) w/ FA (18:1)	triacylglycerols
TG(18:1_33:2)	Triacylglceride (51:3) w/ FA (18:1)	triacylglycerols
TG(18:1_33:3)	Triacylglceride (51:4) w/ FA (18:1)	triacylglycerols
TG(18:1_34:1)	Triacylglceride (52:2) w/ FA (18:1)	triacylglycerols

TG(18:1_34:2)	Triacylglceride (52:3) w/ FA (18:1)	triacylglycerols
TG(18:1_34:3)	Triacylglceride (52:4) w/ FA (18:1)	triacylglycerols
TG(18:1_34:4)	Triacylglceride (52:5) w/ FA (18:1)	triacylglycerols
TG(18:1_35:2)	Triacylglceride (53:3) w/ FA (18:1)	triacylglycerols
TG(18:1_35:3)	Triacylglceride (53:4) w/ FA (18:1)	triacylglycerols
TG(18:1_36:0)	Triacylglceride (54:1) w/ FA (18:1)	triacylglycerols
TG(18:1_36:1)	Triacylglceride (54:2) w/ FA (18:1)	triacylglycerols
TG(18:1_36:2)	Triacylglceride (54:3) w/ FA (18:1)	triacylglycerols
TG(18:1_36:3)	Triacylglceride (54:4) w/ FA (18:1)	triacylglycerols
TG(18:1_36:4)	Triacylglceride (54:5) w/ FA (18:1)	triacylglycerols
TG(18:1_36:5)	Triacylglceride (54:6) w/ FA (18:1)	triacylglycerols
TG(18:1_36:6)	Triacylglceride (54:7) w/ FA (18:1)	triacylglycerols
TG(18:1_38:5)	Triacylglceride (56:6) w/ FA (18:1)	triacylglycerols
TG(18:1_38:6)	Triacylglceride (56:7) w/ FA (18:1)	triacylglycerols
TG(18:1_38:7)	Triacylglceride (56:8) w/ FA (18:1)	triacylglycerols
TG(18:2_28:0)	Triacylglceride (46:2) w/ FA (18:2)	triacylglycerols
TG(18:2_30:0)	Triacylglceride (48:2) w/ FA (18:2)	triacylglycerols
TG(18:2_30:1)	Triacylglceride (48:3) w/ FA (18:2)	triacylglycerols
TG(18:2_31:0)	Triacylglceride (49:2) w/ FA (18:2)	triacylglycerols
TG(18:2_32:0)	Triacylglceride (50:2) w/ FA (18:2)	triacylglycerols
TG(18:2_32:1)	Triacylglceride (50:3) w/ FA (18:2)	triacylglycerols
TG(18:2_32:2)	Triacylglceride (50:4) w/ FA (18:2)	triacylglycerols
TG(18:2_33:0)	Triacylglceride (51:2) w/ FA (18:2)	triacylglycerols
TG(18:2_33:1)	Triacylglceride (51:3) w/ FA (18:2)	triacylglycerols
TG(18:2_33:2)	Triacylglceride (51:4) w/ FA (18:2)	triacylglycerols
TG(18:2_34:0)	Triacylglceride (52:2) w/ FA (18:2)	triacylglycerols
TG(18:2_34:1)	Triacylglceride (52:3) w/ FA (18:2)	triacylglycerols
TG(18:2_34:2)	Triacylglceride (52:4) w/ FA (18:2)	triacylglycerols
TG(18:2_34:3)	Triacylglceride (52:5) w/ FA (18:2)	triacylglycerols
TG(18:2_34:4)	Triacylglceride (52:6) w/ FA (18:2)	triacylglycerols
TG(18:2_35:1)	Triacylglceride (53:3) w/ FA (18:2)	triacylglycerols
TG(18:2_35:2)	Triacylglceride (53:4) w/ FA (18:2)	triacylglycerols
TG(18:2_35:3)	Triacylglceride (53:5) w/ FA (18:2)	triacylglycerols
TG(18:2_36:0)	Triacylglceride (54:2) w/ FA (18:2)	triacylglycerols
TG(18:2_36:1)	Triacylglceride (54:3) w/ FA (18:2)	triacylglycerols
TG(18:2_36:2)	Triacylglceride (54:4) w/ FA (18:2)	triacylglycerols
TG(18:2_36:3)	Triacylglceride (54:5) w/ FA (18:2)	triacylglycerols
TG(18:2_36:4)	Triacylglceride (54:6) w/ FA (18:2)	triacylglycerols
TG(18:2_36:5)	Triacylglceride (54:7) w/ FA (18:2)	triacylglycerols
TG(18:2_38:4)	Triacylglceride (56:6) w/ FA (18:2)	triacylglycerols
TG(18:2_38:5)	Triacylglceride (56:7) w/ FA (18:2)	triacylglycerols
TG(18:2_38:6)	Triacylglceride (56:8) w/ FA (18:2)	triacylglycerols
TG(18:3_30:0)	Triacylglceride (48:3) w/ FA (18:3)	triacylglycerols
TG(18:3_32:0)	Triacylglceride (50:3) w/ FA (18:3)	triacylglycerols
TG(18:3_32:1)	Triacylglceride (50:4) w/ FA (18:3)	triacylglycerols
TG(18:3_33:2)	Triacylglceride (51:5) w/ FA (18:3)	triacylglycerols
TG(18:3_34:0)	Triacylglceride (52:3) w/ FA (18:3)	triacylglycerols

TG(18:3_34:1)	Triacylglceride (52:4) w/ FA (18:3)	triacylglycerols
TG(18:3_34:2)	Triacylglceride (52:5) w/ FA (18:3)	triacylglycerols
TG(18:3_34:3)	Triacylglceride (52:6) w/ FA (18:3)	triacylglycerols
TG(18:3_35:2)	Triacylglceride (53:5) w/ FA (18:3)	triacylglycerols
TG(18:3_36:1)	Triacylglceride (54:4) w/ FA (18:3)	triacylglycerols
TG(18:3_36:2)	Triacylglceride (54:5) w/ FA (18:3)	triacylglycerols
TG(18:3_36:3)	Triacylglceride (54:6) w/ FA (18:3)	triacylglycerols
TG(18:3_36:4)	Triacylglceride (54:7) w/ FA (18:3)	triacylglycerols
TG(18:3_38:5)	Triacylglceride (56:8) w/ FA (18:3)	triacylglycerols
TG(18:3_38:6)	Triacylglceride (56:9) w/ FA (18:3)	triacylglycerols
TG(20:0_32:3)	Triacylglceride (52:3) w/ FA (20:0)	triacylglycerols
TG(20:0_32:4)	Triacylglceride (52:4) w/ FA (20:0)	triacylglycerols
TG(20:0_34:1)	Triacylglceride (54:1) w/ FA (20:0)	triacylglycerols
TG(20:1_24:3)	Triacylglceride (44:4) w/ FA (20:1)	triacylglycerols
TG(20:1_26:1)	Triacylglceride (46:2) w/ FA (20:1)	triacylglycerols
TG(20:1_30:1)	Triacylglceride (50:2) w/ FA (20:1)	triacylglycerols
TG(20:1_31:0)	Triacylglceride (51:1) w/ FA (20:1)	triacylglycerols
TG(20:1_32:1)	Triacylglceride (52:2) w/ FA (20:1)	triacylglycerols
TG(20:1_32:2)	Triacylglceride (52:3) w/ FA (20:1)	triacylglycerols
TG(20:1_32:3)	Triacylglceride (52:4) w/ FA (20:1)	triacylglycerols
TG(20:1_34:0)	Triacylglceride (54:1) w/ FA (20:1)	triacylglycerols
TG(20:1_34:1)	Triacylglceride (54:2) w/ FA (20:1)	triacylglycerols
TG(20:1_34:2)	Triacylglceride (54:3) w/ FA (20:1)	triacylglycerols
TG(20:1_34:3)	Triacylglceride (54:4) w/ FA (20:1)	triacylglycerols
TG(20:2_32:0)	Triacylglceride (52:2) w/ FA (20:2)	triacylglycerols
TG(20:2_32:1)	Triacylglceride (52:3) w/ FA (20:2)	triacylglycerols
TG(20:2_34:1)	Triacylglceride (54:3) w/ FA (20:2)	triacylglycerols
TG(20:2_34:2)	Triacylglceride (54:4) w/ FA (20:2)	triacylglycerols
TG(20:2_34:3)	Triacylglceride (54:5) w/ FA (20:2)	triacylglycerols
TG(20:2_34:4)	Triacylglceride (54:6) w/ FA (20:2)	triacylglycerols
TG(20:2_36:5)	Triacylglceride (56:7) w/ FA (20:2)	triacylglycerols
TG(20:3_32:0)	Triacylglceride (52:3) w/ FA (20:3)	triacylglycerols
TG(20:3_32:1)	Triacylglceride (52:4) w/ FA (20:3)	triacylglycerols
TG(20:3_32:2)	Triacylglceride (52:5) w/ FA (20:3)	triacylglycerols
TG(20:3_34:0)	Triacylglceride (54:3) w/ FA (20:3)	triacylglycerols
TG(20:3_34:1)	Triacylglceride (54:4) w/ FA (20:3)	triacylglycerols
TG(20:3_34:2)	Triacylglceride (54:5) w/ FA (20:3)	triacylglycerols
TG(20:3_34:3)	Triacylglceride (54:6) w/ FA (20:3)	triacylglycerols
TG(20:3_36:3)	Triacylglceride (56:6) w/ FA (20:3)	triacylglycerols
TG(20:3_36:4)	Triacylglceride (56:7) w/ FA (20:3)	triacylglycerols
TG(20:3_36:5)	Triacylglceride (56:8) w/ FA (20:3)	triacylglycerols
TG(20:4_30:0)	Triacylglceride (50:4) w/ FA (20:4)	triacylglycerols
TG(20:4_32:0)	Triacylglceride (52:4) w/ FA (20:4)	triacylglycerols
TG(20:4_32:1)	Triacylglceride (52:5) w/ FA (20:4)	triacylglycerols
TG(20:4_32:2)	Triacylglceride (52:6) w/ FA (20:4)	triacylglycerols
TG(20:4_33:2)	Triacylglceride (53:6) w/ FA (20:4)	triacylglycerols
TG(20:4_34:0)	Triacylglceride (54:4) w/ FA (20:4)	triacylglycerols

TG(20:4_34:1)	Triacylglceride (54:5) w/ FA (20:4)	triacylglycerols
TG(20:4_34:2)	Triacylglceride (54:6) w/ FA (20:4)	triacylglycerols
TG(20:4_34:3)	Triacylglceride (54:7) w/ FA (20:4)	triacylglycerols
TG(20:4_35:3)	Triacylglceride (55:7) w/ FA (20:4)	triacylglycerols
TG(20:4_36:2)	Triacylglceride (56:6) w/ FA (20:4)	triacylglycerols
TG(20:4_36:3)	Triacylglceride (56:7) w/ FA (20:4)	triacylglycerols
TG(20:4_36:4)	Triacylglceride (56:8) w/ FA (20:4)	triacylglycerols
TG(20:4_36:5)	Triacylglceride (56:9) w/ FA (20:4)	triacylglycerols
TG(20:5_34:0)	Triacylglceride (54:5) w/ FA (20:5)	triacylglycerols
TG(20:5_34:1)	Triacylglceride (54:6) w/ FA (20:5)	triacylglycerols
TG(20:5_34:2)	Triacylglceride (54:7) w/ FA (20:5)	triacylglycerols
TG(20:5_36:2)	Triacylglceride (56:7) w/ FA (20:5)	triacylglycerols
TG(20:5_36:3)	Triacylglceride (56:8) w/ FA (20:5)	triacylglycerols
TG(22:0_32:4)	Triacylglceride (54:4) w/ FA (22:0)	triacylglycerols
TG(22:1_32:5)	Triacylglceride (54:6) w/ FA (22:1)	triacylglycerols
TG(22:2_32:4)	Triacylglceride (54:6) w/ FA (22:2)	triacylglycerols
TG(22:3_30:2)	Triacylglceride (52:5) w/ FA (22:3)	triacylglycerols
TG(22:4_32:0)	Triacylglceride (54:4) w/ FA (22:4)	triacylglycerols
TG(22:4_32:2)	Triacylglceride (54:6) w/ FA (22:4)	triacylglycerols
TG(22:4_34:2)	Triacylglceride (56:6) w/ FA (22:4)	triacylglycerols
TG(22:5_32:0)	Triacylglceride (54:5) w/ FA (22:5)	triacylglycerols
TG(22:5_32:1)	Triacylglceride (54:6) w/ FA (22:5)	triacylglycerols
TG(22:5_34:1)	Triacylglceride (56:6) w/ FA (22:5)	triacylglycerols
TG(22:5_34:2)	Triacylglceride (56:7) w/ FA (22:5)	triacylglycerols
TG(22:5_34:3)	Triacylglceridev(56:8) w/ FA (22:5)	triacylglycerols
TG(22:6_32:0)	Triacylglceride (54:6) w/ FA (22:6)	triacylglycerols
TG(22:6_32:1)	Triacylglceride (54:7) w/ FA (22:6)	triacylglycerols
TG(22:6_34:1)	Triacylglceride (56:7) w/ FA (22:6)	triacylglycerols
TG(22:6_34:2)	Triacylglceride (56:8) w/ FA (22:6)	triacylglycerols
TG(22:6_34:3)	Triacylglceride (56:9) w/ FA (22:6)	triacylglycerols
Cer(d16:1/18:0)	Ceramide (d16:1/18:0)	ceramides
Cer(d16:1/20:0)	Ceramide (d16:1/20:0)	ceramides
Cer(d16:1/22:0)	Ceramide (d16:1/22:0)	ceramides
Cer(d16:1/23:0)	Ceramide (d16:1/23:0)	ceramides
Cer(d16:1/24:0)	Ceramide (d16:1/24:0)	ceramides
Cer(d18:1/14:0)	Ceramide (d18:1/14:0)	ceramides
Cer(d18:1/16:0)	Ceramide (d18:1/16:0)	ceramides
Cer(d18:1/18:0 (OH))	Hydroxyceramide (d18:1/18:0(OH))	ceramides
Cer(d18:1/18:0)	Ceramide (d18:1/18:0)	ceramides
Cer(d18:1/18:1)	Ceramide (d18:1/18:1)	ceramides
Cer(d18:1/20:0(OH))	Hydroxyceramide (d18:1/20:0(OH))	ceramides
Cer(d18:1/20:0)	Ceramide (d18:1/20:0)	ceramides
Cer(d18:1/22:0)	Ceramide (d18:1/22:0)	ceramides
Cer(d18:1/23:0)	Ceramide (d18:1/23:0)	ceramides
Cer(d18:1/24:0)	Ceramide (d18:1/24:0)	ceramides
Cer(d18:1/24:1)	Ceramide (d18:1/24:1)	ceramides
Cer(d18:1/25:0)	Ceramide (d18:1/25:0)	ceramides

Cer(d18:1/26:0)	Ceramide (d18:1/26:0)	ceramides
Cer(d18:1/26:1)	Ceramide (d18:1/26:1)	ceramides
Cer(d18:2/14:0)	Ceramide (d18:2/14:0)	ceramides
Cer(d18:2/16:0)	Ceramide (d18:2/16:0)	ceramides
Cer(d18:2/18:0)	Ceramide (d18:2/18:0)	ceramides
Cer(d18:2/18:1)	Ceramide (d18:2/18:1)	ceramides
Cer(d18:2/20:0)	Ceramide (d18:2/20:0)	ceramides
Cer(d18:2/22:0)	Ceramide (d18:2/22:0)	ceramides
Cer(d18:2/23:0)	Ceramide (d18:2/23:0)	ceramides
Cer(d18:2/24:0)	Ceramide (d18:2/24:0)	ceramides
Cer(d18:2/24:1)	Ceramide (d18:2/24:1)	ceramides
Cer(d18:0/18:0 (OH))	Hydroxydihydrocer- amide (d18:0/18:0(OH))	dihydroceramides
Cer(d18:0/18:0)	Dihydroceramide (d18:0/18:0)	dihydroceramides
Cer(d18:0/20:0)	Dihydroceramide (d18:0/20:0)	dihydroceramides
Cer(d18:0/22:0)	Dihydroceramide (d18:0/22:0)	dihydroceramides
Cer(d18:0/24:0)	Dihydroceramide (d18:0/24:0)	dihydroceramides
Cer(d18:0/24:1)	Dihydroceramide (d18:0/24:1)	dihydroceramides
Cer(d18:0/26:1 (OH))	Hydroxydihydrocer- amide (d18:0/26:1(OH))	dihydroceramides
Cer(d18:0/26:1)	Dihydroceramide (d18:0/26:1)	dihydroceramides
